# The effect of a structured running exercise intervention on non-exercise physical activity and sedentary behaviour in persons with mild Multiple Sclerosis and healthy controls

**DOI:** 10.1186/s44167-023-00037-1

**Published:** 2023-12-04

**Authors:** Ine Nieste, Jan Spaas, Wouter M. A. Franssen, Paul V. Asch, Hans H. C. M. Savelberg, Bert O. Eijnde

**Affiliations:** 1 SMRC Sports Medicine Research Center, BIOMED, Biomedical Research Institute, Faculty of Medicine and Life Sciences, Hasselt University, Hasselt, Belgium; 2 Department of Nutrition and Movement Sciences, NUTRIM, School for Nutrition and Translation Research Maastricht, Faculty of Health, Medicine and Life Sciences, Maastricht University, Maastricht, The Netherlands; 3 University MS Center (UMSC) Hasselt, Pelt, Hasselt, Belgium; 4 Department of Movement and Sports Sciences, Faculty of Medicine and Health Sciences, Ghent University, Ghent, Belgium; 5 REVAL, Rehabilitation Research Center, Faculty of Rehabilitation Sciences, Hasselt University, Hasselt, Belgium; 6 Move to Sport Foundation, Kontich, Belgium; 7 Division of Sport Science, Faculty of Medicine and Health Sciences, Stellenbosch University, Stellenbosch, South Africa

**Keywords:** Multiple Sclerosis, Exercise, Non-exercise physical activity, Sedentary behaviour, Exercise adaptations

## Abstract

**Background:**

Exercise interventions fail to increase objective physical activity (PA) in persons with Multiple Sclerosis (PwMS), while they self-report higher exercise participation. This suggests that PwMS change their non-exercise PA (NEPA). We aimed to explore NEPA changes of PwMS and healthy controls (HC), and whether these constrain exercise adaptations.

**Methods:**

Twenty-nine mildly-disabled PwMS and 26 HC completed a 10-month home-based running program. A non-randomised controlled study design was used. The primary outcome was time in different NEPA intensities (light intensity PA [LIPA] and moderate-to-vigorous intensity PA [MVPA]) and in sedentary behaviour ([SB]; total and uninterrupted SB) at baseline (T1), after 5 (T2) and 10 (T3) months of exercise. Data were averaged over days with and without exercise sessions (EX and NONEX days). Secondary outcomes included patient-reported and physical exercise adaptations (fatigue, walking mobility, blood pressure, body composition and cardiorespiratory fitness).

**Results:**

A significant reduction in non-exercise MVPA was observed from T1 to T2 (− 113 ± 31 min/week, p < 0.01) and from T1 to T3 (− 95 ± 26 min/week, p < 0.01) in PwMS, which approximately matched the weekly exercise duration at those time points. PwMS also increased their uninterrupted SB on NONEX days compared to EX days (+ 0.7 ± 0.3 h, p < 0.01). There were no changes in MVPA or SB of HC (group × time effect MVPA: p < 0.05; group × EX day effect uninterrupted SB: p < 0.01). Secondary outcomes improved similarly in both groups and were not associated with NEPA/SB changes.

**Conclusions:**

In contrast to HC, PwMS significantly changed their NEPA and the pattern in which they accumulated SB in response to structured exercise. This might be a necessary behavioural compensation in order to adhere to the exercise intervention and did not constrain patient-reported and physical outcomes. Future research is warranted to unravel the underlying causes and to investigate the effects on other exercise adaptations, such as cardiometabolic health.

*Trial registration* The present study was registered (December 10, 2019) at clinicaltrials.gov as NCT04191772

**Supplementary Information:**

The online version contains supplementary material available at 10.1186/s44167-023-00037-1.

## Background

Multiple sclerosis (MS) is an autoimmune, inflammatory and neurodegenerative disorder of the central nervous system, predominantly affecting young to middle-aged adults [1]. Persons with MS (PwMS) manifest with heterogeneous symptoms, commonly including spasticity, paralysis, walking difficulties, fatigue and cognitive decline [2]. Because of the aforementioned disease symptoms, PwMS are often more sedentary and less active compared to healthy controls (HC) [3, 4]. To date, a plethora of evidence shows the beneficial effects of structured exercise on MS symptoms, cardiorespiratory fitness and the risk of developing comorbidities that are associated with disability and disease progression, such as obesity and hypertension [5–8]. Consequently, strategies to increase long-term participation in structured exercise have been well studied in PwMS. The results show that important perceived barriers, such as lack of time, transportation, accessibility and specialist availability, can be overcome by implementing home-based exercise, which is feasible, safe and beneficial for PwMS [9].

However, it is currently not known what happens with the non-exercise PA (NEPA) of PwMS in response to exercise. Changes in NEPA, defined as unstructured and unplanned PA embedded in much of daily life, such as climbing stairs, doing household chores and active transportation [10], can be hypothesised to impact the eventual exercise adaptations. Moreover, previous observational work in patient populations (i.e. Parkinson’s disease and older adults with disabilities in activities of daily living) showed that NEPA levels and structured vigorous-intensity exercise have independent effects on health outcomes and disability [11, 12]. This highlights the relevance of investigating NEPA changes in response to an exercise intervention to further optimise rehabilitation outcomes. Furthermore, results of previous systematic reviews and a meta-analysis indicate that there is a discrepancy between objectively measured PA and self-reported exercise participation in PwMS following an exercise intervention, with only the latter showing effects [13, 14]. Coote et al., whose results were included in both reviews, hypothesised that PwMS might reduce their NEPA in order to engage in exercise training [15], but did not measure this during the intervention. Hence, specific NEPA changes and the effect on the eventual exercise adaptations, are not known yet.

More specifically, the lack of an objective PA increase [13, 14] might be caused by a reduction in the intensity of NEPA (i.e. a shift from moderate-to-vigorous intensity to more light intensity PA), or by an increase in sedentary behaviour (SB). SB is defined as “any waking behaviour in a sitting, reclining or lying posture with an energy expenditure ≤ 1.5 metabolic equivalents” [16]. These changes might have a distinct impact on the eventual exercise adaptations. Furthermore, previous research did not include healthy controls (HC), thereby limiting conclusions on whether NEPA/SB changes are MS-specific or rather a consequence of the exercise protocol. Therefore, the present study primarily aimed to investigate the specific changes in daily NEPA/SB of PwMS and HC during a home-based running exercise intervention, and secondarily whether these changes are associated with the eventual exercise adaptations in patient-reported outcomes (fatigue and walking disability) and physical outcomes (blood pressure, body composition and cardiorespiratory fitness).

## Methods

### Study design

The exercise intervention consisted of a 10-month home-based running exercise program (February 2020–December 2020). The study design comprised a non-randomised controlled study. Because there is no data available on NEPA changes in PwMS during an exercise intervention, sample size calculation was based on the change in cardiorespiratory fitness previously reported by our research group with a similar exercise protocol [17]. An estimated effect size of 0.5 resulted in 42 PwMS and 42 HC needed to detect a significant change of 5.2% from pre- to post-intervention with a power of 80% and a two-sided α using a paired t-test and drop-out rate of 10%.

After checking the eligibility of participants via mail or phone, participants were invited for the first study visit to assess baseline measurements (T1) (see Additional file 1). Primary outcome measures included NEPA on days with and without exercise sessions (EX and NONEX days), and were monitored by accelerometry for 24 h/day during 7 consecutive days at T1, after 5 months of intervention (T2), and in the last week of the intervention (T3). Secondary outcome measures included patient-reported outcomes (fatigue and walking impairment) and physical outcomes (blood pressure, body composition and cardiorespiratory fitness), and were measured at T1 and T3 (after the intervention).

### Participants

PwMS and HC (> 18 years) were recruited through online and paper advertisements via the Belgian-based MS foundation MoveToSport (Kontich, Belgium). PwMS were included if they had mild disability (Expanded Disability Status Scale score; EDSS < 5), independent of the MS phenotype. Participants were excluded if they experienced an acute MS exacerbation 6 months prior to the start of the study, did not receive written medical clearance from their general practitioner to participate in moderate-to-vigorous intensity PA (MVPA), had medication changes in the last three months, planned or were planning to follow a weight reduction program or weight loss (> 2 kg) in the last three months before study enrolment (i.e. stable or non-changing dietary habits and physical activity patterns) in order to prevent interference with exercise effects on body composition measures, were pregnant, or had no daily internet access. All participants provided written informed consent prior to participation. The study protocol was approved by the Medical Ethical Committee of Hasselt University (Hasselt, Belgium; CME2019/062) and the Jessa Hospital Hasselt (Belgium), was conducted in accordance with the principles of the Declaration of Helsinki (2013) and is registered at clinicaltrials.gov as NCT04191772 (December 19, 2019).

### Exercise program

The intervention consisted of running, as this requires minimal equipment and no transportation, to facilitate the implementation of a long-term aerobic exercise program in a home-based context. Furthermore, adherence to running instructions can be objectively measured with a sports watch, as recommended by previous research [18]. More specifically, running instructions were communicated weekly via a Polar^®^ M430 sports watch (Polar Electro Oy, Finland) and were based on the maximal heart rate (HR_max_) measured during a cycle exercise test (see ‘secondary outcome measures’), increased by 5% to correct for the transfer between cycling and running [19]. Based on baseline cardiorespiratory fitness (reference values from Heyward et al. [20]) and running experience, participants were assigned to either a start to run (STR) or an experienced run (ER) program (see Additional file 1). In the STR program, participants alternated walking and running in the first 3 months until they could run continuously for 30 min. Hereafter, STR participants progressed to the ER program. The design of both programs was periodised, as this has previously been shown by our research group to exert superior cardiorespiratory fitness outcomes compared to classic, moderate-intensity endurance training [21]. Both programs comprised periodically alternating blocks of 3 weeks. The first two weeks of every cycle included one high intensity training ([HIIT]; 20–30 min, 80–100%HR_max_) and two moderate intensity continuous training ([MICT]; 30–80 min, 60–80% HR_max_) sessions/week. The third week of every cycle comprised only one HIIT or two MICT sessions, alternately, to allow recuperation. Each exercise session started and ended with a 5-min warm-up/cool-down (50–60% HR_max_) period (see Additional file 2 for more details).

### Measurements

#### Primary outcome measures: NEPA and SB

A tri-axial activPAL3^®^ monitor (PAL Technologies, Glasgow, Scotland) was used to quantify NEPA and SB for 24 h/day during 7 consecutive days. The monitor was waterproofed with a small sleeve and medical-grade adhesive wrapping (Tegaderm; 3 M, Saint Paul, MN, USA) and attached on the mid part of the anterior thigh of participants using Tegaderm (3 M). The proprietary algorithm in the activPAL software was used to generate second-by-second estimates of total PA and SB. The following outcome measures were used to describe NEPA and SB:

- Light intensity PA ([LIPA]; stand + walking at < 99 steps/min [22]), in % of waking time (WT)/day
- Moderate-to-vigorous intensity PA ([MVPA]; walking, running and cycling at ≥ 100 steps/min [22]), in % of WT/day
- Total SB (% of WT/day)
- Uninterrupted SB (time in sedentary bouts > 60 min; hours)

These outcomes were averaged for:

- All measured days without an exercise session (NONEX days), and
- All measured days with an exercise session (EX days), in which exercise time was removed from the raw data generated by the activPAL algorithm.

Data were only included when there were at least 4 measurement days of 24 h/day with a minimum of 1 weekend day and 2 exercise sessions (for T2 and T3). WT was determined by subtracting sleeping time (determined by the activPAL algorithm and manually checked/corrected with self-reported time-in-bed data [23]) from the total wear time/day.

#### Secondary outcome measures

##### Exercise session information

Participants tracked their exercise sessions (intensity in % of HR_max_ and duration in minutes) with a sports watch and uploaded this information via an online Polar account. Exercise session information was then available for the research team via polar.flow.com/coach. Session adherence was calculated as the number of completed exercise sessions compared to the protocol (% of the total prescribed sessions) [18], and content adherence as the total training duration and time per heart rate zone compared to the protocol (% of the prescribed total duration/time per heart rate zone) [18].

##### Patient-reported outcomes

Multidimensional (physical, cognitive and psychosocial) fatigue of the last four weeks was assessed by the Modified Fatigue Impact Scale (MFIS) [24]. Total (range 0–84) and different MFIS dimension scores were reported. In addition, the number of participants with a total MFIS score ≥ 38, which is the cut-off value for MS-related fatigue [25], was reported.

The impact of MS on walking mobility was measured by the 12-item MS Walking Scale (MSWS-12), for which higher scores indicate worse perceived walking ability [0–100] [26].

##### Physical outcomes

After sitting on a chair for 10 min in a quiet room with constant temperature (21 °C), HR_rest_ (beats per minute; bpm), systolic and diastolic blood pressure (SBP and DBP; mm Hg) were assessed four times at 5-min intervals with an electronic sphygmomanometer (Omron^®^, Omron Healthcare, IL, USA) on the dominant arm and documented as the mean value of the final 3 measurements.

Body height was measured to the nearest 0.1 cm using a wall-mounted Harpenden stadiometer, with participants barefoot. Body weight (BW; kg) was determined using a digital-balanced weighting scale in underwear to the nearest 0.1 kg. Body mass index (BMI) was calculated from weight and height measurements (weight/height^2^). Whole body fat percentage (fat%; %) and lean tissue mass (kg) were evaluated using a dual energy X-ray absorptiometry (Hologic Series Delphi-A Fan Beam X-ray Bone Densitometer, Vilvoorde, Belgium).

A gradual maximal exercise test was performed on an electronically braked cycle ergometer (eBike Basic^®^, General Electric GmbH, Bitz, Germany), which provides a valid measure of cardiorespiratory fitness in PwMS with low to mild levels of disability (EDSS ≤ 4.0) [27]. Participants started cycling at 20 (♀) or 30 (**♂**) watt (W) for 1 min, followed by workload increases of 10 or 15 W/min respectively, while maintaining a cadence of > 70 rounds per minute (rpm). HR was monitored continuously using a Polar chest strap (Polar^®^, Oy, Finland), while oxygen uptake (V̇O_2_), expiratory volume and respiratory exchange ratio (RER) were measured breath-by-breath and averaged every 10 s using a Metalyzer II^®^B (Cortex, Leipzig, Germany). Exercise testing was terminated by volitional exhaustion or failure to maintain 45 rpm. The RER (≥ 1.10), maximal heart rate (HR_max_; ≥ 90% of age-predicted HR [220-age]), post-test lactate levels (2 min after termination; ≥ 8 mmol/L) and perceived exertion (Borg scale; ≥ 17/20) were used to verify maximal effort. Peak oxygen uptake (V̇O_2peak_) relative to total body mass (ml/min/kg), peak workload (W_peak_) and the HR recovery 2 min after termination (HR_recov_; % of HR_max_) were reported. In addition, V̇O_2peak_ values were compared with the predicted V̇O_2peak_ values based on age, sex and body weight [28] and presented as %V̇O_2peakPRED_.

### Statistical analyses

Statistical analyses were performed using IBM SPSS^®^ version 27.0 (IBM SPSS Statistics for Windows, Chicago, IL, USA). Participant characteristics, primary outcome measures and exercise session information were presented as means ± standard error of the mean (SEM), exercise adaptations as estimated means ± SEM. Data were analysed using a per-protocol (PP) principle, for which all participants with a session adherence < 70% were excluded. After checking the assumptions, baseline differences between groups were checked with a univariate analysis of variance (with sex as covariate for the secondary outcome measures). PA data were analysed using a linear mixed model analysis for repeated measures including main and interaction effects for time (T1, T2, T3), group (MS, HC) and exercise day (EX, NONEX day), participant ID as random effect and an unstructured repeated covariance structure for the residuals. The same model was used for the secondary outcome measures, without the factor for exercise day and the addition of sex as covariate. The model validation was performed with likelihood-ration tests. When a significant main effect was found, Bonferroni-corrected pairwise comparisons were performed. Exercise information was analysed using a univariate analysis of variance with group (MS, HC) as fixed factor. The categorical data were compared between groups (MS, HC) with a chi-squared test. To investigate whether NEPA changes impacted the secondary outcome measures, partial correlations between NEPA changes (T1 to T3) and changes in secondary outcomes were made for the whole group, corrected for baseline values and sex. Correction for multiple testing was implemented using the Benjamini–Hochberg false discovery rate (FDR) method [29]. To explore explanatory factors for the NEPA changes, partial correlations between NEPA changes (T1 to T3) and demographics (age, EDSS, MS duration), baseline secondary outcomes (total and physical fatigue, walking mobility, V̇O_2peak_, fat%) and exercise information (exercise protocol and duration) were made for the whole group, and PwMS only. A p-value ≤ 0.05 (2-tailed) was considered statistically significant for all analyses.

## Results

### Participant characteristics

**Fig. 1:**
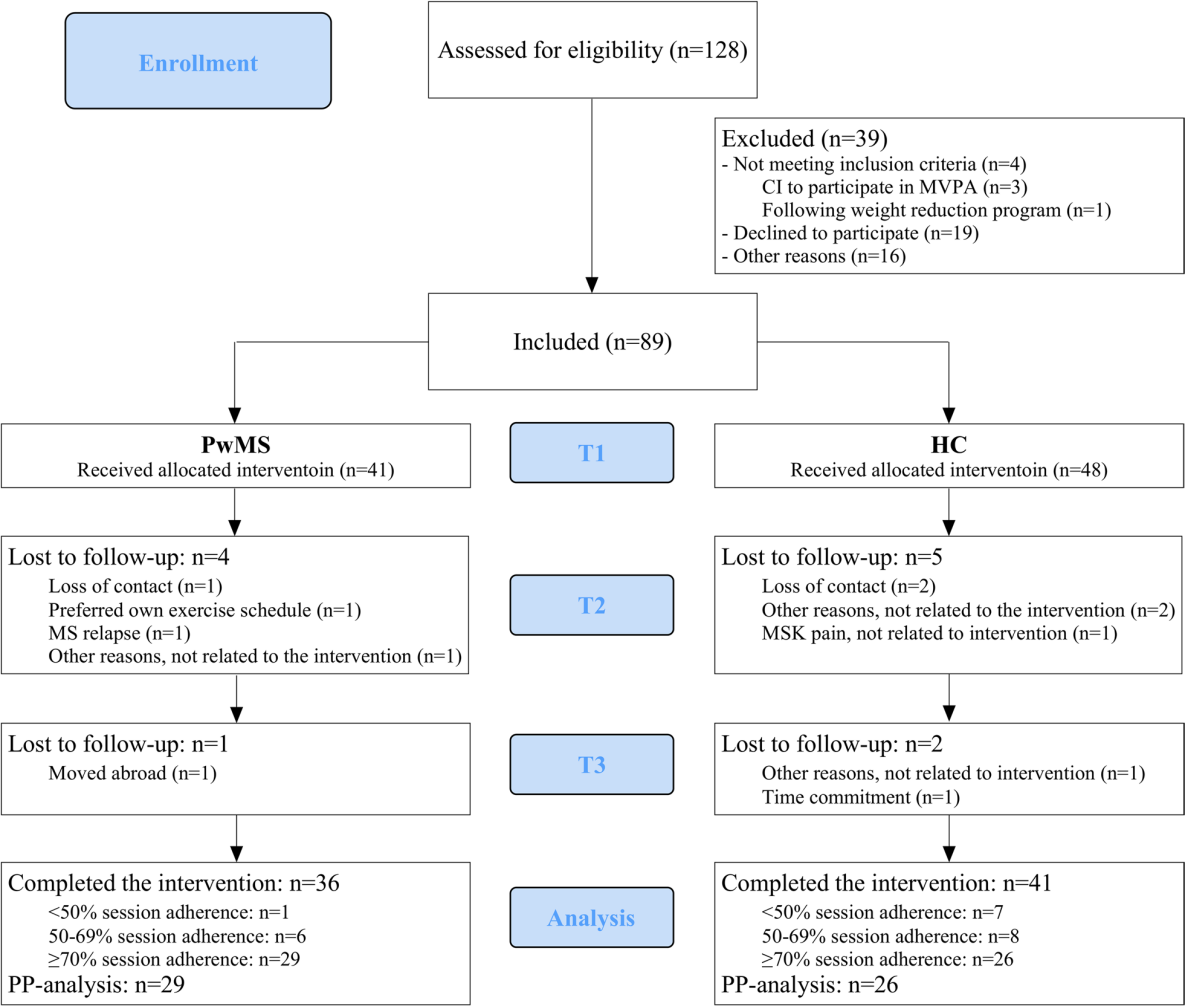
Flow chart of study participants. **CI** Contra-indications, **MVPA** moderate-to-vigorous intensity physical activity, **PwMS** persons with Multiple Sclerosis, **HC** healthy controls, **MSK** musculoskeletal, **PP** per-protocol

A total of 128 participants were screened for study eligibility, of which 89 were included (Fig. 1). All participants (41 PwMS, 48 HC) completed the baseline measurements, after which 5 HC and 4 PwMS dropped out at T2 and another 2 HC and 1 PwMS at T3, resulting in 36 PwMS and 41 HC completing the intervention. Because the aim of the current study was to explore PA changes of PwMS who performed exercise consistently, only the data of participants with ≥ 70% session adherence (29 PwMS and 26 HC) are presented here. There were no differences in baseline measures or participant characteristics between participants with < 70% or ≥ 70% adherence. Participants were on average 41.3 ± 1.0 years and the majority was female (PwMS: 86.2%, HC: 65.4%, p = 0.111). All PwMS had the relapsing–remitting phenotype, a mean MS duration of 8.2 ± 1.2 years at study enrolment and were only mildly disabled (mean EDSS score 1.4 ± 0.2) (Table 1). A higher percentage of PwMS performed the STR protocol compared to the HC (37.9% *vs.* 11.5%, p = 0.032).

**Table 1:** Participant characteristics

	PwMS (n = 29)	HC (n = 26)	p-values
Gender (female, %)	86.2	65.4	0.111
Age (year)	41.3 ± 1.4	41.2 ± 1.5	0.969
BMI (kg/m^2^)	24.9 ± 0.6	24.6 ± 0.7	0.730
Mean EDSS score (0–4)	1.4 ± 0.2	–	–
EDSS score range (0–4)	0–4		
MS duration (y)	8.2 ± 1.2	–	–
Time since relapse (y)	4.2 ± 0.7	–	–
Comorbidities (%)			
DMT2	0	0	–
CVD	6.9	3.8	1.000
Pulmonary disease	0	0	–
Thyroid disease	10.3	3.8	0.613
Smokers (%)	3.4	0	1.000

Data are expressed as means ± SEM. **PwMS** persons with Multiple Sclerosis, **HC** healthy controls, **BMI** body mass index, **EDSS** expanded disability status, **DMT2** diabetes mellitus type 2, **CVD** cardiovascular diseases

### Primary outcome measures: NEPA and SB

**Fig. 2:**
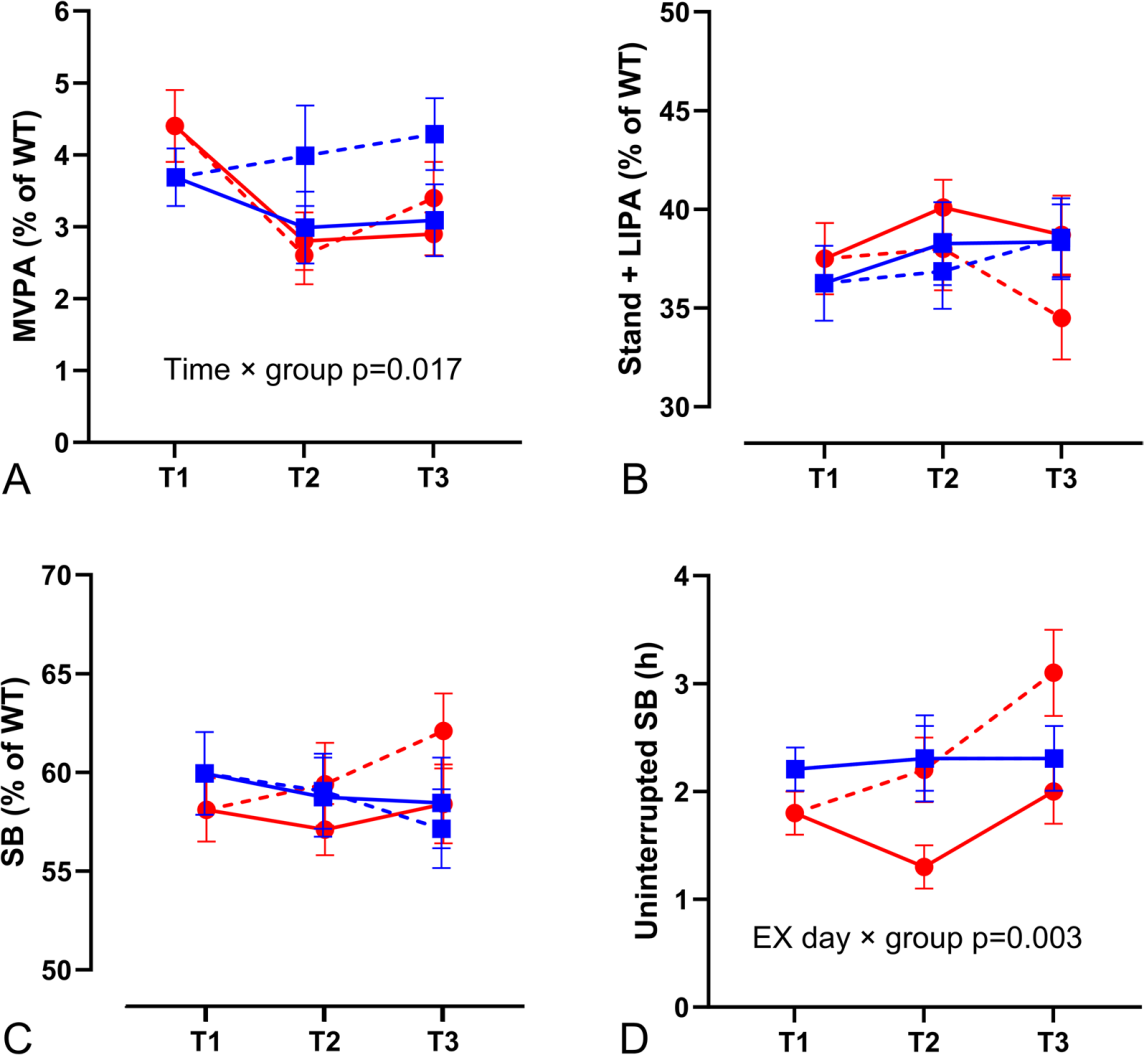
Non-exercise physical activity and sedentary behaviour. Solid lines present exercise days, dashed lines non-exercise days at T1 (before start of the intervention), T2 (5 months within the intervention), and T3 (last week of the intervention). PwMS are presented in red (red circle), HC in blue (blue square). **A** Moderate-to-vigorous intensity physical activity (**MVPA**), **B** light intensity physical activity (**LIPA**; stand + light intensity walking), and **C** sedentary behaviour (**SB**) are presented in % of waking time (**WT**) (without exercise time for EX days), **D** uninterrupted SB (time in sedentary bouts > 60 min) in hours (h)/day. All data are expressed as means ± SEM. An overview with the absolute data and exact p-values can be found in Additional file 3

Besides a longer sleep duration in PwMS (+ 0.6 ± 0.2 h, p = 0.001; group effect p < 0.001), which did not change during the intervention (interaction effects p > 0.05; see Additional file 3), all other baseline PA and SB characteristics were similar between groups. On both T2 and T3, PwMS significantly reduced their MVPA levels compared to T1 (− 1.6 ± 0.5% of WT/day or − 113 ± 31 min/week, p = 0.006 and − 1.3 ± 0.4% of WT/day or − 95 ± 26 min/week, p = 0.007 respectively), whilst this did not change in HC (− 0.1 ± 0.6% of WT/day and + 0.1 ± 0.5% of WT/day, p = 1.000; group × time interaction effect p = 0.017; Fig. 2 and Additional file 3). Of the PwMS reducing their MVPA, approximately 50% replaced it with LIPA and 50% with SB. These changes were not statistically significant. Furthermore, PwMS increased their uninterrupted SB on NONEX compared to EX days (+ 0.7 ± 0.3 h, p < 0.001), while there was no difference between EX and NONEX days in HC (− 0.1 ± 0.2 h, p = 0.907; group × EX day interaction effect p = 0.003; Fig. 2). There were no significant correlations between changes in NEPA or SB and demographics, baseline secondary outcomes nor exercise protocol.

### Secondary outcome measures

#### Exercise session information

**Fig. 3:**
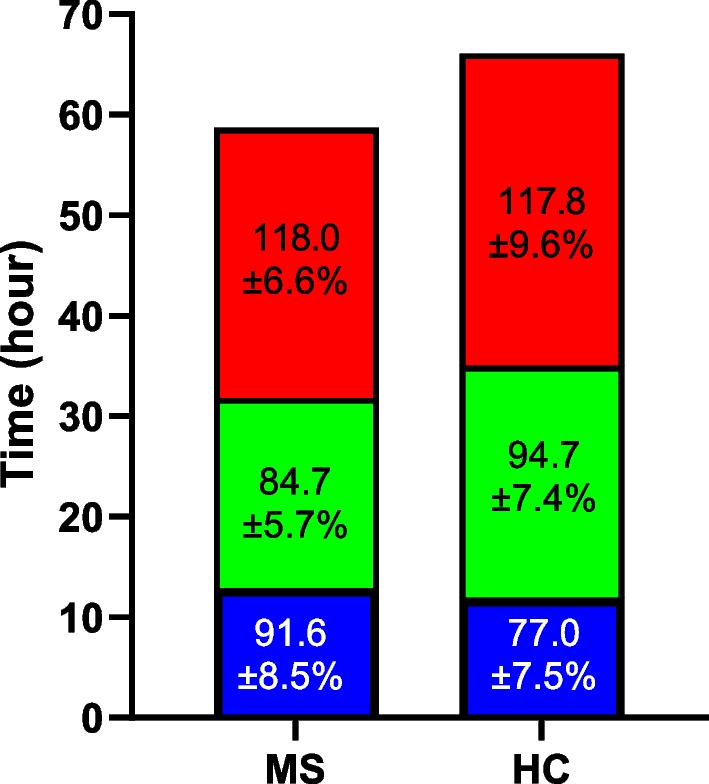
Time in hour per heart rate zone. Blue rectangular box 50–70%, Green rectangular box 70–80%, Red rectangular box 80–100% of HR_max_. Percentages depicted in the bars present content adherence: % of total duration/time per heart rate zone according to the protocol (p > 0.05 between groups). **MS** Multiple Sclerosis, **HC **healthy controls. Data are expressed as means ± SEM

The mean exercise duration per session was longer for HC (44 ± 1 min *vs*. 41 ± 1 min; p = 0.031), but there were no differences in the total number of exercise sessions (89 ± 2 sessions in 10 months, p = 0.221), mean exercise intensity (78.7 ± 0.8% of HR_max_, p = 0.116), session adherence (92.4 ± 1.8% p = 0.234), nor content adherence (94.9 ± 2.0%, p = 0.167 and Fig. 3) between groups. PwMS and HC also did not differ in total exercise time performed during the 1-week T2 and T3 PA assessment (T2 PwMS: 114 ± 9 min, HC: 104 ± 8 min, p = 0.424 and T3 PwMS: 105 ± 9 min, HC: 130 ± 9 min, p = 0.053).

#### Exercise adaptations

At baseline, W_peak_ and cardiorespiratory fitness (V̇O_2peak_ and V̇O_2peakPRED_) were significantly lower in PwMS than HC and more PwMS reached the cut-off value of MS-related fatigue. These differences between groups did not change after the intervention. Fatigue (− 5.8 ± 1.2 points), fat% (− 1.0 ± 0.4%), HR_rest_ (− 3 ± 1 bpm), W_peak_ (+ 12.3 ± 1.6W), cardiorespiratory fitness (V̇O_2peak_ + 2.1 ± 0.5 ml/min/kg) and HR_recov_ (+ 1.7 ± 0.6%) improved similarly in both groups (see Table 2 for p-values of time effects). No significant associations were observed between changes in NEPA/SB and changes in secondary outcome measures.

**Table 2:** Exercise adaptations

	PwMS (n = 29)		HC (n = 26)		p-values		
	T1	T3	T1	T3	Time	Group	Time x group
PRO							
MFIS (0–84)	30.8 ± 2.7	24.5 ± 2.5	24.1 ± 2.8	18.9 ± 2.6	**< 0.001**	0.082	0.836
Cog (0–40)	15.7 ± 1.5	13.7 ± 1.6	12.8 ± 1.6	11.7 ± 1.6	**0.042**	0.215	0.779
Phys (0–36)	12.7 ± 1.3	9.3 ± 1.2	9.7 ± 1.3	6.2 ± 1.2	**< 0.001**	0.060	0.953
PS (0–8)	2.3 ± 0.3	1.5 ± 0.3	1.7 ± 0.3	1.2 ± 0.3	**0.003**	0.151	0.476
MFIS ≥ 38 (n, %)	11/28 (39.3)^^^	9/29 (31.0)*	2/26 (7.7)^^^	0/24 (0.0)*	**–**	–	–
MSWS -12 (0–100)	26.6 ± 1.5	25.5 ± 1.8	–	–	0.393	–	–
**Physical outcomes**							
SBP (mmHg)	117 ± 2	116 ± 2	117 ± 3	115 ± 2	0.243	0.948	0.466
DBP (mmHg)	73 ± 2	75 ± 1	74 ± 2	74 ± 2	0.409	0.957	0.493
HR_rest_ (bpm)	70 ± 2	67 ± 2	68 ± 2	64 ± 2	**< 0.001**	0.358	0.362
LM (kg)	43.2 ± 1.0	43.5 ± 1.0	44.2 ± 1.0	44.1 ± 1.0	0.540	0.564	0.406
FAT% (%)	32.1 ± 1.3	31.2 ± 1.1	29.3 ± 1.3	28.3 ± 1.2	**0.007**	0.103	0.880
W_peak_ (W)	174.5 ± 4.9^	185.6 ± 5.3	193.5 ± 5.2^^^	207.0 ± 5.6	**< 0.001**	**0.009**	0.468
V̇O_2peak_ (ml/min/kg)	34.0 ± 1.1^^^	35.7 ± 1.1	36.9 ± 1.2^^^	39.2 ± 1.1	**< 0.001**	**0.038**	0.518
%V̇O_2peakPRED_ (%)	101.7 ± 2.8^^^	107.2 ± 3.1	112.6 ± 3.0^^^	117.9 ± 3.3	**< 0.001**	**0.012**	0.956
HR_recov_ (%)	23.0 ± 1.2	24.8 ± 1.3	24.9 ± 1.2	26.4 ± 1.3	**0.012**	0.300	0.841

Data are expressed as estimated means ± SEM. ^^^Significant difference between groups at baseline, *significant difference between groups post-intervention. **PwMS** Persons with Multiple Sclerosis, **HC** healthy controls, **T1** before start of the intervention, **T3** after the intervention, **PRO** patient-reported outcomes, **MFIS** modified fatigue impact scale, **Cog** cognitive subscale of MFIS, **Phys** physical subscale of MFIS, **PS** psychosocial subscale of MFIS, **MSWS-12** Multiple Sclerosis walking scale, **SBP** systolic blood pressure, **DBP** diastolic blood pressure, **HR **heart rate, **bpm** beats per minute, **LM** lean mass, **W**_peak_ workload at V̇O_2peak_, **V̇O**_2peak_ peak oxygen uptake, **%V̇O**_2peakPRED_ percentage of predicted V̇O_2peak_, **HR**_recov_ HR recovery

## Discussion

Structured exercise training is a cornerstone of MS treatment, but its effect on NEPA levels of PwMS is unclear. The present data show that PwMS change their NEPA in response to exercise. More specifically, a reduction in non-exercise MVPA was observed after 5 and 10 months (− 113 ± 31 min/week and − 95 ± 26 min/week), which approximately matched the weekly duration of the exercise sessions at those time points (114 ± 9 min/week and 105 ± 9 min/week). This explains why previous studies found no change in total PA levels after an exercise intervention, while PwMS self-reported to do more exercise [13, 14]. Furthermore, PwMS also increased their uninterrupted SB on days without an exercise session compared to days with an exercise session (+ 0.7 ± 0.3 h). These NEPA/SB changes were not seen in HC in the present findings, which is in line with previous findings of systematic reviews in healthy adults and overweight/obese persons [30, 31]. This indicates that the presently measured NEPA changes were MS-specific.

It is intriguing to speculate why the present NEPA changes seem to be MS-specific. Firstly, there were no associations between any of the MS characteristics (EDSS score, MS duration and walking mobility) and NEPA/SB changes. It is, however, important to note that this might also be due to the low variation within the MS characteristics, as the included PwMS were only mildly disabled and had a rather short MS duration. Secondly, fatigue, reported to be the most frequent and debilitating MS symptom [32], was proposed by King et al*.* as an important explanatory factor for inter-individual variability in NEPA changes in healthy persons [33]. In the PwMS of the present study, a large variation in fatigue was measured (range MFIS questionnaire: 2–64 points), but no correlations were present between fatigue at any time point and NEPA or SB changes. Furthermore, fatigue significantly improved with 20.5% in the MS group. This implies that fatigue did not cause the NEPA/SB changes. However, although the MFIS questionnaire assesses multidimensional fatigue, it only captures the average fatigue of the last four weeks. This limits conclusions on acute fatigue and especially on exercise-induced fatigue/tiredness. Smith et al*.* reported that fatigue does not increase immediately nor 24 h after exercise in PwMS, but only a single exercise session was included and the intensity was rather low (Borg scale 12–14/20)[34]. Moreover, an observational study on symptomatic fatigue in PwMS (n = 309) stated that 64% of participants reported worse fatigue after “moderate exercise”, while this was the case for 83% after “vigorous exercise” [35]. Although sub analyses of the current results showed that exercise intensity (HIIT versus MICT sessions) had no effect on the NEPA/SB changes, the overall demand of 2–3 exercise sessions per week at moderate-to-vigorous intensity might require necessary NEPA/SB compensations in order to conserve energy to adhere to the exercise intervention. Such energy conservation strategies are often taught to PwMS to prevent or treat fatigue [36]. However, it might also be true that PwMS implemented dysfunctional energy conservation strategies, which were not adapted according to their improving fatigue and cardiorespiratory fitness levels. This was not investigated in the present study, but this indicates that fatigue and the implementation of energy conservation strategies might need to be monitored and possibly revised throughout an exercise intervention.

Another plausible explanation might be that the current PwMS could not maintain their baseline PA levels in addition to doing more exercise, because their baseline PA was already high (mean ± SD: 41 ± 23 min MVPA/day and 9782 ± 2569 steps/day [data not shown]). However, PwMS in previous research had clearly lower baseline PA levels (mean ± SD: 26 ± 18 min MVPA/day, 4488 ± 2251 steps/day and 6095 ± 2363 steps/day), but their total PA also did not increase while they self-reported to do more exercise [13, 14]. In a study of Keadle et al. [37], overweight/obese participants had even higher baseline MVPA (mean ± SD 50 ± 17 min/day) than the present PwMS, and they were able to further increase this during an exercise intervention when they also received education on SB and self-monitoring of non-exercise MVPA. This might also be possible in PwMS when they receive a multicomponent intervention targeting both exercise PA and NEPA/SB and should be explored in future research.

The last important factor to take into consideration, is that the PA assessments at T2 and T3 were conducted during the COVID-19 pandemic. Although both assessments occurred during periods with only minor COVID-related restrictions in Belgium, it could be hypothesised that PwMS reduced their social and/or outdoor related PA to a greater extent compared to HC due to the autoimmune nature of their disease. Pedullà et al*.* indeed showed in a large international study that there was a 10% reduction in the number of PwMS meeting the recommended PA guidelines during the pandemic, independent of disability [38]. However, PA decreases are also reported for HC in international reports [39], and there are no studies available yet comparing pandemic-related PA changes between PwMS and HC. Furthermore, Pedullà et al*.* also showed that mildly disabled PwMS only reduced their physical therapy at the clinic and exercise at the gym, while walking and running remained the same as pre-pandemic [38]. Because the present intervention consisted of running, it can be assumed that our findings were a consequence of the intervention rather than the pandemic. In addition, our results were similar at two different time points, and also in line with previous studies in both PwMS [13, 14] and HC [30, 31] that were conducted before the COVID-19 pandemic.

To the best of our knowledge, this was the first running intervention program of longer duration in PwMS. Feys et al. previously implemented a running intervention, but this only lasted 3 months and PwMS only reached the maximal running dosage in the final weeks [40]. Both session and content adherence were high in the PwMS in the present PP-analysis (90.4 ± 2.6% and 92.2 ± 2.9%), but also in the total sample (n = 36; 82.9 ± 3.6% and 84.7 ± 3.8% [data not shown]), indicating that the present exercise program is a feasible intervention for home-based exercise for PwMS. Furthermore, our findings are in line with previous studies including different exercise modalities [13, 14], suggesting that the presently measured NEPA/SB compensations are independent of the exercise modality. The exercise intervention effectively improved the cardiorespiratory fitness, body composition, fatigue and resting HR in both groups, with no difference between groups. This is in line with previous periodised exercise interventions in PwMS and HC [17, 21]. However, this might seem contradictory, because PwMS changed their NEPA and SB whilst HC did not. Moreover, the non-exercise MVPA reduction approximately matched the exercise time, which would result in an unaffected net MVPA change. The fact that exercise effects were not constrained, might be explained by a difference in PA intensity. More specifically, the exercise intensity was probably higher (78.7 ± 6.1% of HR_max_) compared to that of the reduced non-exercise MVPA, limiting the impact on exercise outcomes. Unfortunately, it is not possible to draw strong conclusions on the exact intensity of the reduced NEPA, because all walking and running activities with ≥ 100 steps/min are classified as MVPA by the activPAL algorithm. It might be relevant to include continuous heart rate monitoring in future research in order to measure the exact NEPA intensities.

Additionally, the present PwMS were only mildly disabled and already very physically fit at baseline (on average > 100% of their predicted cardiorespiratory fitness). It could be hypothesised that more disabled or less fit PwMS show larger NEPA/SB compensations in response to exercise, which may in turn have negative effects on the eventual exercise adaptations. Furthermore, other relevant physical exercise outcomes that were not measured, might have been impacted. In a study of Keadle et al. [41], the effect of changes in NEPA in overweight/obese participants was also assessed on fasting lipids and insulin sensitivity. Interestingly, insulin sensitivity only improved within participants who performed exercise and also reduced their SB (-5%; which was replaced with MVPA and LIPA), not in participants who only performed exercise (with no changes in NEPA/SB). It might be especially relevant to monitor the effect of NEPA and SB changes on insulin sensitivity in PwMS in future research, because they already have a higher risk of developing insulin resistance (× 2.48 compared to HC) [42], which is associated with a worsening of disability [8, 42, 43]. Furthermore, the current findings also show that PwMS significantly increased their uninterrupted SB in response to exercise. This has already been shown to be negatively associated with insulin sensitivity in a large sample of HC (n = 4935), independent of total MVPA and SB [44]. Hence, measures of insulin sensitivity should be included in future NEPA research in PwMS.

The present study has several strengths. Firstly, PA and SB were objectively assessed for seven consecutive days on three different time points throughout the study, and a distinction was made between days with and without exercise. Secondly, the Polar watches provided objective adherence rates in a home-based setting, which enabled a PP-analysis with participants who trained consistently. This allows us to conclude that the observed NEPA/SB changes occur when PwMS effectively perform the prescribed exercise sessions. Furthermore, a post-hoc power calculation showed a similar effect size for the observed improvement in cardiorespiratory fitness as what was a priori calculated (although the eventual sample size was smaller). Lastly, this is the first study that compared NEPA changes between PwMS and HC. There was no randomisation in the present study, but our findings, in probably very motivated PwMS, highlight the relevance of further research to NEPA/SB changes with more rigorous study designs. Finally, the intervention consisted of a running program, which limits the generalisation of results to more disabled PwMS with walking impairments. Future research on NEPA changes in more deconditioned and/or disabled PwMS with other (non-weight bearing) intervention types such as (recumbent) cycling is warranted.

## Conclusion and implications for practice

PwMS reduced their weekly non-exercise MVPA with a duration approximately matching the exercise duration, and they accumulated their SB in longer bouts on days without exercise session. This might be a necessary MS-specific, behavioural compensation in order to adhere to the exercise intervention and did not constrain patient-reported and physical outcomes. Future research is warranted to unravel the underlying causes and to investigate the effects on other exercise adaptations, such as cardiometabolic health.

Based on the present findings, strategies to maintain or even improve NEPA/SB (e.g. education and self-monitoring) should be implemented in future exercise interventions. Furthermore, it might be useful to assess fatigue after exercise sessions and its effect on NEPA/SB on both EX and NONEX days, in order to adapt exercise interventions accordingly and individually. Lastly, the implementation of energy conserving techniques should be monitored and adequately revised throughout the exercise intervention.

## Supplementary Information

**Additional file 1.** Study design.

**Additional file 2.** Exercise programs.

**Additional file 3.** Non-exercise physical activity and sedentary behaviour on exercise and non-exercise days.

## Data Availability

The datasets used and/or analysed during the current study are stored in a permanent repository and are available from the corresponding author on reasonable request.

## Abbreviations

BMI: Body mass index
BPM: Beats per minute
BW: Body weight
CI: Contra-indications
CVD: Cardiovascular diseases
DBP: Diastolic blood pressure
DMT2: Diabetes mellitus type 2
EDSS: Expanded disability status scale score
ER program: Experienced run program
EX days: Days with exercise session
HC: Healthy controls
HIIT: High intensity interval training
HR: Heart rate
HR_max_: Maximal heart rate
HR_recov_: Heart rate recovery
HR_rest_: Resting heart rate
LIPA: Light intensity physical activity
LM: Lean mass
MFIS: Modified fatigue impact scale
MFIS Cog: Cognitive subscale of Modified fatigue impact scale
MFIS Phys: Physical subscale of Modified fatigue impact scale
MFIS PS: Psychosocial subscale of Modified fatigue impact scale
MICT: Moderate intensity continuous training
MS: Multiple Sclerosis
MSK: Musculoskeletal
MSWS-12: 12-Item MS walking scale
MVPA: Moderate-to-vigorous intensity physical activity
NEPA: Non-exercise physical activity
NONEX days: Days without exercise session
PA: Physical activity
PP: Per protocol
PRO: Patient-reported outcomes
PwMS: Persons with Multiple Sclerosis
RER: Respiratory exchange ratio
RPM: Rounds per minute
SB: Sedentary behaviour
SBP: Systolic blood pressure
SEM: Standard error of the mean
STR program: Start to run program
V̇O_2peak_: Peak oxygen uptake
V̇O_2peakPRED_: Predicted peak oxygen uptake
W_peak_: Peak workload
WT: Waking time
