# Supplementary material for: The effect of a structured running exercise intervention on non-exercise physical activity and sedentary behaviour in persons with mild Multiple Sclerosis and healthy controls
Source: J Act Sedentary Sleep Behav. 2023 Dec 4;2:29. doi: 10.1186/s44167-023-00037-1 (PMC11960282; doi:10.1186/s44167-023-00037-1)
Supplement: Supplementary file 1 — Additional file 1. Study design. [file 44167_2023_37_MOESM1_ESM.docx]

**Additional file 1**

**Study design.** Secondary outcome measures (resting heart rate, blood pressure, fatigue [MFIS; Modified Fatigue Impact Scale], walking impairment [MSWS-12; 12-item MS Walking Scale], body composition and cardiorespiratory fitness; CRF) were assessed at T1 before the start of the intervention (week -2) and at T3 after completion of the intervention (week 45). Primary outcome measures (non-exercise physical activity/sedentary behaviour) were performed at T1 (week -1), T2 (5 months; week 22) and T3 (week 44) with an activPAL monitor. The intervention comprised a 10-month home-based Start To Run (STR) or Experienced Run (ER) exercise program, which was based on baseline CRF (reference values from Heyward et al.^1^) and running experience; STR = poor, fair and good CRF without running experience; ER = fair and good CRF with running experience, excellent and superior CRF.

**References**

1. Heyward V. Advanced fitness assessment and exercise prescription: Human kinetics Champaign. IL, 2006.
