# Supplementary material for: The effect of a structured running exercise intervention on non-exercise physical activity and sedentary behaviour in persons with mild Multiple Sclerosis and healthy controls
Source: J Act Sedentary Sleep Behav. 2023 Dec 4;2:29. doi: 10.1186/s44167-023-00037-1 (PMC11960282; doi:10.1186/s44167-023-00037-1)
Supplement: Supplementary file 2 — Additional file 2. Exercise programs. [file 44167_2023_37_MOESM2_ESM.docx]

| Exercise programs | | | | | | | | | | | | |
| --- | --- | --- | --- | --- | --- | --- | --- | --- | --- | --- | --- | --- |
| **Intensity (% of HR_max_)** | | | **60-70%** | | **70-80%** | | **80-90%** | | **90-100%** | | **Total** | |
|  | **Week** |  | **STR** | **ER** | **STR** | **ER** | **STR** | **ER** | **STR** | **ER** | **STR** | **ER** |
| **Cycle 1** | 1-2 | HIIT | **7x3’** | 3x5’ | **7x2’** |  |  | 3x1’ |  |  | 45’ | 28’ |
|  |  | MICT 1 | **8x4’** | 2x2’ | **8x1’** | 2x10’ |  |  |  |  | 50’ | 34’ |
|  |  | MICT 2 | **50’** | 20’ |  |  |  |  |  |  | 60’ | 30’ |
|  | 3 | HIIT | **3x5’** | 3x5’ |  |  | **3x1’** |  |  | 3x20’’ | 28’ | 26’ |
|  |  | *Optional* |  |  |  | *35’* |  |  |  |  |  | 45’ |
| **Cycle 2** | 4-5 | HIIT | **5x3’** | 3x5’ | **5x3’** |  |  | 3x1’ |  |  | 40’ | 28’ |
|  |  | MICT 1 | **6x2’** | 2x2’ | **6x3’** | 2x15’ |  |  |  |  | 40’ | 44’ |
|  |  | MICT 2 | **7x3’** |  | **7x2’** | 20’ |  |  |  |  | 45’ | 30’ |
|  | 6 | MICT 3 | **3x4’** |  |  | 30’ | **3x1’** |  |  |  | 25’ | 40’ |
|  |  | MICT 4 |  |  |  | 30’ |  |  |  |  |  | 40’ |
| **Cycle 3** | 7-8 | HIIT | **3x3’** | 3x3’ | **3x4’** |  |  |  |  | 3x20’’ | 31’ | 20’ |
|  |  | MICT 1 | **4x3’** | 4x3’ | **4x3’** |  |  | 4x1’ |  |  | 34’ | 26’ |
|  |  | MICT 2 | **4x4’** |  | **4x4’** | 40’ |  |  |  |  | 42’ | 50’ |
|  | 9 | HIIT | **4x5’** | 4x3’ |  |  | **4x1’** |  |  | 4x20’’ | 34’ | 23,3’ |
|  |  | *Optional* |  |  |  | *45’* |  |  |  |  |  | 55’ |
| **Cycle 4** | 10-11 | HIIT | **3x2’** | 4x3’ | **3x4’** |  |  | 4x30’’ |  |  | 28’ | 24’ |
|  |  | MICT 1 | **5x2’** | 5x3’ | **5x3’** |  |  | 5x1’ |  |  | 35’ | 30’ |
|  |  | MICT 2 | **3x4’** |  | **3x5’** | 50’ |  |  |  |  | 37’ | 60’ |
|  | 12 | MICT 3 | **2x2’** |  | **2x10’** | 55’ |  |  |  |  | 34’ | 65’ |
|  |  | MICT 4 |  |  |  | 55’ |  |  |  |  |  | 65’ |
| **Cycle 5** | 13-14 | HIIT | **3x4’** | 4x3’ |  |  | **3x1’** |  |  | 4x30’’ | 25’ | 24’ |
|  |  | MICT 1 | **2x2’** | 3x4’ | **2x10’** |  |  | 3x2’ |  |  | 34’ | 28’ |
|  |  | MICT 2 |  | 60’ | **20’** |  |  |  |  |  | 30’ | 70’ |
|  | 15 | HIIT | **3x5’** | 4x3’ |  |  |  |  | **3x20’’** | 4x30’’ | 24’ | 24’ |
|  |  | *Optional* |  |  | ***25’*** | *60’* |  |  |  |  | 35’ | 70’ |
| **Cycle 6** | 16-17 | HIIT | **4x4’** | 4x2’ |  |  | **4x1’** |  |  | 4x30’’ | 30’ | 20’ |
|  |  | MICT 1 | **2x2’** | 3x3’ | **2x12’** |  |  | 3x2’ |  |  | 38’ | 25’ |
|  |  | MICT 2 |  | 2x25’ | **25’** |  |  |  |  | 2x30’’ | 35’ | 61’ |
|  | 18 | MICT 3 |  | 3x2’ | **30’** |  |  | 3x5’ |  |  | 40’ | 31’ |
|  |  | MICT 4 |  | 3x2’ | **30’** |  |  | 3x5’ |  |  | 40’ | 31’ |
| **Cycle 7** | 19-20 | HIIT | **4x3’** | 4x3’ |  |  | **4x1’** |  |  | 4x30’’ | 26’ | 24’ |
|  |  | MICT 1 | **2x2’** | 4x3’ | **2x15’** |  |  | 4x3’ |  |  | 44’ | 34’ |
|  |  | MICT 2 |  |  | **35’** | 60’ |  |  |  |  | 45’ | 70’ |
|  | 21 | HIIT | **3x5’** | 5x3’ |  |  |  |  | **3x30’’** | 5x30’’ | 26,5’ | 27,5’ |
|  |  | *Optional* |  | *70’* | ***40’*** |  |  |  |  |  | 50’ | 80’ |
| **Cycle 8** | 22-23  34-35  43-44 | HIIT | **3x3’** | 4x5’ |  |  |  |  | **3x30’’** | 4x30’’ | 20,5’ | 32’ |
|  |  | MICT 1 | **3x4’** | 4x3’ |  |  | **3x2’** | 4x4’ |  |  | 28’ | 38’ |
|  |  | MICT 2 |  |  | **40’** | 65’ |  |  |  |  | 50’ | 75’ |
|  | 24  36 | MICT 3 |  | 65’ | **35’** |  |  |  |  |  | 45’ | 75’ |
|  |  | MICT 4 |  | 65’ | **35’** |  |  |  |  |  | 45’ | 75’ |
| **Cycle 9** | 25-26  31-32  40-41 | HIIT | **3x2’** | 3x5’ |  |  |  |  | **3x30’’** | 3x40’’ | 17,5’ | 27’ |
|  |  | MICT 1 | **3x3’** | 4x4’ |  |  | **3x3’** | 4x4’ |  |  | 28’ | 42’ |
|  |  | MICT 2 | **2x20’** | 2x30’ |  |  |  | 2x2’ | **2x30’’** |  | 51’ | 74’ |
|  | 27  33 42 | HIIT | **2x3’** | 4x5’ |  |  |  |  | **3x30’’** | 4x40’’ | 17,5’ | 32,7’ |
|  |  | *Optional* | ***40’*** | 65’ |  |  |  |  |  |  | 50’ | 75’ |
| **Cycle 10** | 28-29  37-38 | HIIT | **4x2’** | 4x4’ |  |  |  |  | **4x30’’** | 4x40’’ | 20’ | 28,7’ |
|  |  | MICT 1 | **3x2’** | 4x2’ |  |  | **3x4’** | 4x5’ |  |  | 28’ | 38’ |
|  |  | MICT 2 | **45’** |  |  | 70’ |  |  |  |  | 55’ | 80’ |
|  | 30  39 | MICT 3 | **3x4’** |  |  | 50’ | **3x4’** |  |  |  | 34’ | 60’ |
|  |  | MICT 4 | **3x4’** |  |  | 50’ | **3x4’** |  |  |  | 34’ | 60’ |
| Data represent the amount of time per heart rate zone (% of the maximal heart rate; **HR_max_**) in minutes (‘) and seconds (’’). **STR** Start To Run program, **ER** Experienced Run program, **HIIT** High-Intensity Interval Training, **MICT** low-to moderate-intensity continuous training. Exercise bouts from different intensities were alternated. 5 min warm-up/cool down periods at 50-60% HR_max_ are not showed and need to be added to the total exercise duration. In the STR program, 60-70% HR_max_ zones from cycle 1🡪4 comprised walking and from cycle 5🡪10 running. After week 30, cycli 8, 9 and 10 were repeated in both programs. | | | | | | | | | | | | |

**Additional file 2**
