# Supplementary material for: The effect of a structured running exercise intervention on non-exercise physical activity and sedentary behaviour in persons with mild Multiple Sclerosis and healthy controls
Source: J Act Sedentary Sleep Behav. 2023 Dec 4;2:29. doi: 10.1186/s44167-023-00037-1 (PMC11960282; doi:10.1186/s44167-023-00037-1)
Supplement: Supplementary file 3 — Additional file 3. Non-exercise physical activity and sedentary behaviour on exercise and non-exercise days. [file 44167_2023_37_MOESM3_ESM.docx]

**Additional file 3**

| Non-exercise physical activity and sedentary behaviour on exercise and non-exercise days | | | | | | | | | | | | | |  | |
| --- | --- | --- | --- | --- | --- | --- | --- | --- | --- | --- | --- | --- | --- | --- | --- |
|  | **PwMS (n: 28)** | | | **HC (n: 22)** | | | | **P-values** | | | | | |  | |
|  | **T1** | **T2** | **T3** | | **T1** | **T2** | **T3** | **Time** | **EX day** | **Group** | **Time  x EX day** | **Time  x group** | **EX day x group** | | **Time x EX day  x group** |
| Sleeping time (h)  NONEX day  EX day | 8.5 ± 0.2^ | 8.6 ± 0.2  8.8 ± 0.2 | 8.6 ± 0.2  8.8 ± 0.1 | | 8.1 ± 0.1^ | 7.9 ± 0.2  8.1 ± 0.2 | 8.1 ± 0.1  7.9 ± 0.2 | 0.939 | 0.481 | **<0.001** | 0.558 | 0.699 | 0.306 | | 0.658 |
| MVPA (% of WT*)  NONEX day  EX day | 4.4 ± 0.5 | 2.6 ± 0.4  2.8 ± 0.4 | 3.4 ± 0.5  2.9 ± 0.3 | | 3.7 ± 0.4 | 4.0 ± 0.7  3.0 ± 0.5 | 4.3 ± 0.5  3.1 ± 0.5 | 0.394 | **0.028** | 0.655 | 0.620 | **0.017** | 0.104 | | 0.815 |
| LIPA (% of WT*)  NONEX day  EX day | 37.5 ± 1.8 | 38.0 ± 2.1  40.1 ± 1.4 | 34.5 ± 2.1  38.7 ± 2.0 | | 36.3 ± 1.9 | 36.9 ± 1.9  38.3 ± 2.1 | 38.6 ± 2.0  38.4 ± 1.9 | 0.572 | **0.025** | 0.743 | 0.993 | 0.747 | 0.290 | | 0.520 |
| SB (% of WT*)  NONEX day  EX day | 58.1 ± 1.6 | 59.4 ± 2.1  57.1 ± 1.3 | 62.1 ± 1.9  58.4 ± 2.0 | | 60.0 ± 2.1 | 59.1 ± 1.9  58.7 ± 2.0 | 57.1 ± 2.0  58.5 ± 2.3 | 0.288 | 0.107 | 0.833 | 0.879 | 0.200 | 0.111 | | 0.518 |
| Uninterrupted SB (h)  NONEX day  EX day | 1.8 ± 0.2 | 2.2 ± 0.3  1.3 ± 0.2 | 3.1 ± 0.4  2.0 ± 0.3 | | 2.2 ± 0.2 | 2.3 ± 0.3  2.3 ± 0.4 | 2.3 ± 0.3  2.3 ± 0.3 | **0.004** | 0.003 | 0.270 | 0.540 | 0.111 | **0.003** | | 0.768 |
| Data are expressed as means ± SEM. ^Significant difference between groups at baseline. Abbreviations: **T1** before start of the intervention, **T2** after 5 months during the intervention, **T3** during the last week of the intervention, **NONEX** non-exercise, **EX** exercise, **WT** waking time**, MVPA** moderate-to-vigorous intensity physical activity, **LIPA** light-intensity physical activity, **SB** sedentary behaviour (standing + walking at light-intensity). *without exercise time. | | | | | | | | | | | | | | | |
